# Bi-regional and bi-phasic automated machine learning radiomics for defining metastasis to lesser curvature lymph node stations in gastric cancer

**DOI:** 10.1186/s40644-025-00891-z

**Published:** 2025-06-08

**Authors:** Huilin Huang, Siwen Wang, Jingyu Deng, Zhaoxiang Ye, Hailin Li, Bingxi He, Mengjie Fang, Nannan Zhang, Jiaxin Liu, Di Dong, Han Liang, Guoxin Li, Jie Tian, Yanfeng Hu

**Affiliations:** 1 Department of General Surgery, Guangdong Provincial Key Laboratory of Precision Medicine for Gastrointestinal Tumor, Nanfang Hospital, Southern Medical University, Guangzhou, 510515 Guangdong China; 2 CAS Key Laboratory of Molecular Imaging, Institute of Automation, Chinese Academy of Sciences, Beijing, 100190 China; 3 School of Artificial Intelligence, University of Chinese Academy of Sciences, Beijing, 100049 China; 4 Department of Gastric Cancer Surgery, Key Laboratory of Cancer Prevention and Therapy, Tianjin Medical University Cancer Institute and Hospital, National Clinical Research Center for Cancer, Tianjin’s Clinical Research Center for Cancer, Tianjin, Tianjin, 300060 China; 5 Department of Radiology, Tianjin’s Clinical Research Center for Cancer, Key Laboratory of Cancer Prevention and Therapy, Tianjin Medical University Cancer Institute and Hospital, National Clinical Research Center for Cancer, Tianjin, 300060 China; 6 Beijing Advanced Innovation Center for Big Data-Based Precision Medicine, School of Engineering Medicine, Beihang University, Beijing, 100191 China; 7 State Key Laboratory of Cancer Biology and National Clinical Research Center for Digestive Diseases, Xijing Hospital of Digestive Diseases, Fourth Military Medical University, 710000 Xi’an, China; 8 National Key Laboratory of Kidney Diseases, Beijing, 100853 China; 9 Cancer center of Beijing Tsinghua Changgung Hospital, School of Clinical Medicine, Tsinghua Medicine，Tsinghua University, 102218 Beijing, China

**Keywords:** Stomach neoplasms, Lymphatic metastasis, Radiomics, Tomography, X-Ray computed

## Abstract

**Background:**

Lymph node metastasis (LNM) is the primary metastatic mode in gastric cancer (GC), with frequent occurrences in lesser curvature. This study aims to establish a radiomic model to predict the metastatic status of lymph nodes in the lesser curvature for GC.

**Methods:**

We retrospectively collected data from 939 gastric cancer patients who underwent gastrectomy and D2 lymphadenectomy across two centers. Both the primary lesion and the lesser curvature region were segmented as representative region of interests (ROIs). The combination of bi-regional and bi-phasic CT imaging features were used to build a hybrid radiomic model to predict LNM in the lesser curvature. And the model was validated internally and externally. Further, the potential generalization ability of the hybrid model was investigated in predicting the metastasis status in the supra-pancreatic area.

**Results:**

The hybrid model yielded substantially higher performance with AUCs of 0.847 (95% CI, 0.770–0.924) and 0.833 (95% CI, 0.800-0.867) in the two independent test cohorts, compared to the single regional and phasic models. Additionally, the hybrid model achieved AUCs ranging from 0.678 to 0.761 in the prediction of LNM in supra-pancreatic area, showing the potential generalization performance.

**Conclusions:**

The CT imaging features of primary tumor and adjacent tissues are significantly associated with LNM. And our as-developed model showed great diagnostic performance and might be of great application in the individual treatment of GC.

**Supplementary Information:**

The online version contains supplementary material available at 10.1186/s40644-025-00891-z.

## Background

Lymph node metastasis (LNM) is the main metastatic pathway of gastric cancer (GC) and associated with poor clinical outcomes, including the need for extended resection, high perioperative morbidities, and a heightened risk of recurrence, necessitating more aggressive therapeutic strategy [1–3]. LNM is increasingly valued in the assessment of GC, as patients with LNM should undergo the lymphadenectomy and extended resection [4]. 

The lymphatic drainage from stomach follows anatomy and physiology different pathway along main gastric arteries according to the gastric regions and reaches the celiac nodes [5]. Notably, the region of lesser curvature and supra-pancreas is the initial and terminal drainage field of peri-gastric lymph system, making it particularly prone to LNM [6, 7]. Additionally, regional lymph node (LN) dissection in the supra-pancreas presents significant challenges for surgeons due to the complex vascular variations that involves the common hepatic artery and the splenic artery. Metastatic LNs tend to adhere to the vessels, and the surrounding tissue becomes thickened and indistinct, causing an unclear display and increasing the risk of bleeding [8]. Therefore, monitoring LNM in the lesser curvature and the supra-pancreatic regions is crucial for primary treatment decision-making and preoperative planning [5, 9]. 

Nowadays, there is still no valid non-invasive instrument to diagnose LNM. Computed tomography (CT) is the most commonly utilized tool recommended by the National Comprehensive Cancer Network (NCCN) guidelines [10] to evaluate LN status before operation [11, 12]. The current standard for LNM assessment via CT focus on LN size and axial ratio, with nodes exceeding 6 mm generally considered metastatic [13, 14]. Besides, PET/CT can utilize glucose metabolism to demonstrate superior diagnostic performance over conventional CT in detecting LNM [15]. However, these imaging methods remain constrained by spatial resolution and cannot detect metastatic LNs with diameters of 3 mm or less, which are defined as occult lymph node metastases (OLNM). Studies have revealed that 14.5% of metastatic LNs have a maximum diameter of less than 3 mm. It makes clinical decision-making heavily dependent on the radiologist’s expertise and leading to potential underdiagnosis in CT imaging [16, 17]. 

To increase the diagnostic accuracy, radiomics, a quantitative image analysis technique, is employed to derive features from medical images, thereby describing the most representative region of interests (ROIs) and predicting treatment response [18–23]. Previous studies have successfully applied this technique to predict LNM following neoadjuvant chemotherapy. However, these studies focused on ROIs of either primary lesions or radiologically detectable lymph nodes, thereby overlooking the clinical significance of OLNM [24, 25]. In this study, we delineated both the primary lesion and the lesser curvature region, and attempted to elucidate the most relevant features about LNM. Moreover, we developed and validated a new radiomic model to predict the LN stations status of lesser curvature, and further generalize to predicting LNM in the supra-pancreatic area.

## Methods

### Study population

Inclusion criteria included (1) Our study enrolled patients with GC who underwent gastrectomy with D2 lymphadenectomy. (2) The diagnosis of GC should be confirmed by pathology. (3) The patients performed abdomen contrast-enhanced CT within pre-operation 30 days. (4) The number of LNMs should be equal or more than 16. (5) The status of LNMs were confirmed by pathology and the number of LNs in the lesser curvature should not be less than 1.

Exclusion criteria included (1) The primary lesion cannot be seen in the CT imaging; (2) Patients had the history of previous gastric resection; (3) The clinical information was not available.

A combined size of 939 gastric cancer patients were retrospectively collected from two centers along with corresponding clinicopathological characteristics and image data. Thereinto, 339 consecutive patients at Nanfang Hospital (Guangzhou, China) between August 1st, 2008 and June 30th, 2018 were allocated to a training cohort (*n* = 237) and an internal validation cohort (*n* = 102) at a ratio of 7:3. Patient recruitment criteria are above. With the same enrollment criteria, a total of 600 patients treated at Tianjin Medical University Cancer Institute and Hospital (Tianjin, China) between January 1st, 2016 and September 20th, 2018 were analyzed as an independent external cohort.

The numeric LNs nomenclature origins from Japanese gastric cancer treatment guidelines [4]. It divides the peri-gastric area into 16 regions, depending on the lymphatic drainage. The No.3 station corresponds to lesser curvature while the stations (No. 7, 8, 9, 11) mean the supra-pancreatic area, representing Left Gastric nodes, nodes near proper hepatic artery, Celiac nodes and Nodes near spleen artery respectively.

During surgery, the patient’s LNM status was carefully checked. The No.3 LNM status was defined based on pathological sections stained with hematoxylin and eosin (H&E), and $\:{\text{L}\text{N}\text{M}}_{\text{N}\text{o}.3}^{+}$ denoted LNM and $\:{\text{L}\text{N}\text{M}}_{\text{N}\text{o}.3}^{-}$ represented non-LNM in the lesser curvature. Baseline clinicopathological characteristics were collected from medical records, including age, sex, LNM status in No.3, 7, 8, 9, 11, pathological staging, and clinical report of LNM in No.3.

### Image processing and feature discovery

All patients underwent preoperative contrast-enhanced abdominal CT examination. Arterial phase and portal venous phase CT images were retrieved from the Picture Archiving and Communication System (PACS). The multidetector row CT systems (GE LightSpeed 16, 64-section LightSpeed VCT, GE Medical Systems, Milwaukee, WI, USA; 256-MDCT scanner Brilliance CT, Philips Healthcare, Cleveland, OH, USA) were used to perform contrast-enhanced abdominal CT. After intravenous contrast administration of 90–100 ml iodinated contrast material (Ultravist 370, Bayer Schering Pharma, Berlin, Germany) at a rate of 3.0 or 3.5 ml/s with a pump injector (Ulrich CT Plus 150, Ulrich Medical, Ulm, Germany), arterial and portal venous-phase contrast-enhanced CT images were acquired 28 and 60 s after respectively. The CT acquisition parameters were as below: Tube voltage: 120 kVp; Tube current modulation: Automated dose adjustment with 150–190 mAs reference; Rotation time: 0.4–0.5 s/rot; Detector configuration: 64 × 0.625 mm (GE VCT), 256 × 0.625 mm (Philips iCT); Slice thickness: 5 mm. Contrast-enhanced CT was reconstructed with a reconstruction thickness of 5 mm.

**Fig. 1:**
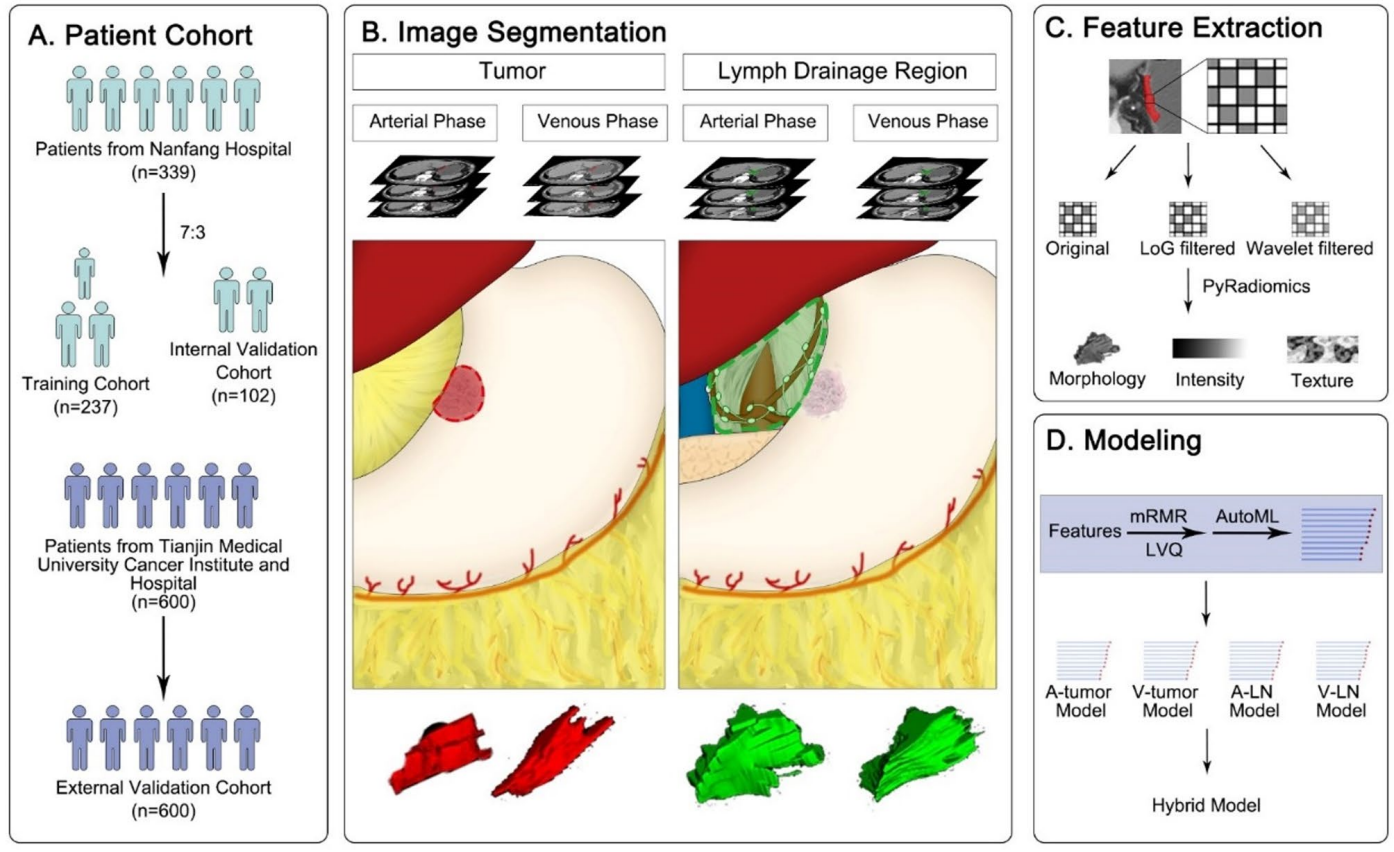
Workflow of this study

The three-dimensional tumor ROI (tumor-ROI) was generated by contouring along the margin of tumors on the image slices with potential tumor area. Also, the No.3 LN ROI (LN-ROI) was delineated on the region of the lesser curvature and supra-pancreas, including all surrounding tissue, because it is difficult to identify alone lesser curvature from the lesser omentum. The segmentation was based on ITK-SNAP (version 3.8, http://www.itksnap.org) and the segmentation method was manual. And two radiologists with 3–5 years of experience joined in the segmentation process and the segmentation results were thereafter validated by two senior radiologists upon consensus. Figure 1 illustrates the overall study design.

The 3D radiomic features were extracted from tumor-ROIs and LN-ROIs based on PyRadiomics (version 3.0.1) in Python (version 3.7, https://www.python.org/). All image slices were interpolated with B-spline interpolation algorithm and had a resampled pixel spacing of 1.0 × 1.0 × 1.0 mm^3^. The gray values were discretized using a fixed bin size of 20 Hounsfield Units. The image types included original images, Laplacian of Gaussian (LoG)-filtered images, and wavelet-filtered images. For LoG filter, the width of Gaussian kernel was set to 1.0, 3.0, and 5.0 mm. For wavelet filter, we adopted Coiflet1 to yield eight decompositions in three dimensions, including HLL, LHL, LLH, HHL, HLH, LHH, HHH, and LLL. Then, a total of 1070 radiomic features were extracted each, including 14 morphology features, 216 intensity features, and 840 texture features.

### Development of bi-regional and bi-phasic radiomic models

We considered combinations of bi-regions and bi-phases, including arterial phase tumor (A-tumor) model, arterial phase LN (A-LN) model, venous phase tumor (V-tumor) model, and venous phase LN (V-LN) model. Radiomic feature selection and model building was performed in the training cohort for these four cases, respectively.

In feature selection, supervised feature ranking and clustering was conducted via minimum redundancy maximum relevance (mRMR) algorithm and learning vector quantization (LVQ) algorithm. The mRMR algorithm ranked features by balancing their mutual information with the No.3 LNM status against inter-feature redundancy. A total of 110 features (approximately 10% of the 1070 extracted features) were retained, following a commonly used heuristic for high-dimensional feature reduction [26]. Subsequently, the LVQ algorithm was employed to rank the retained features by their relevance to the classification task, using one prototype per class and a default learning rate of 0.01 over 100 iterations [27]. To investigate the optimal number of features, we fitted radiomic models based on feature subsets consisting of the top 5, 10, 15, and 20 features from LVQ, respectively.

As to model building, we adopted a fast and lightweight AutoML tool, FLAML [28], which is designed for efficient hyperparameter optimization with low computational cost. FLAML supports multiple learners and integrates adaptive, cost-aware search strategies, including greedy search with iterative refinement, rather than conventional grid or random search. In this study, the candidate models included XGBoost, LightGBM [29], Extra Trees, Random Forest (RF), and logistic regression with L1 regularization (LRL1). For each radiomic model, the AutoML pipeline automatically selected the best-performing learner and tuned its hyperparameters within a time budget of one minute. Five-fold cross-validation was employed internally by FLAML during the optimization process to evaluate model performance, with the area under receiver operating characteristic (ROC) curves (AUC) used as the primary evaluation metric. The hyperparameter search space was adaptively defined based on the selected learner. For boosting-based models (LightGBM and XGBoost), key hyperparameters such as n_estimators (50–1000), max_depth [3–12], learning_rate (0.005–0.2), subsample, and colsample_bytree were tuned using log-uniform or uniform sampling strategies. For ensemble tree models (Extra Trees and RF), FLAML explored hyperparameters including n_estimators, max_depth, min_samples_split, and min_samples_leaf. For (LRL1, the regularization strength C was sampled from a log-uniform distribution ranging from 1e-4 to 1e2. All hyperparameter optimization and model selection were completed within the allocated time budget for each model. Finally, four radiomic models (A-tumor, A-LN, V-tumor, and V-LN), reflecting distinct phenotypes of tumors and lymph nodes across arterial and venous phase CT images, were developed to predict No.3 LNM status. All procedures, including feature selection, model training, and hyperparameter tuning, were implemented using Python-based libraries: pymrmr, sklearn-lvq, and flaml.

### Hybrid model performance evaluation and comparison

We then built a hybrid radiomic model with the combination of A-tumor, A-LN, V-tumor, and V-LN models by using multivariate logistic regression. The classification performances of radiomic models were evaluated in internal validation and external validation cohorts with respect to AUC, sensitivity, specificity, and accuracy. The optimal thresholds were the points closest to the top-left part of the plot with perfect sensitivity or specificity, as defined as follows. The decision curve analysis (DCA) was also carried out to quantify the models’ clinical usefulness.

$$\:Optimality\:criterion:\:{min}{\:(1\:-\:sensitivities)}^{2}+{(1-\:specificities)}^{2}$$

Further, to evaluate the robustness of bi-regional and bi-phasic radiomic models, we conducted repeated random subsampling validation by randomly partitioning the Nanfang Hospital dataset five times and repeating evaluation via Monte Carlo cross-validation. We reported the average model performance. In each partition, the radiomic features were standardized by z-score method using the mean and standard deviation values of the corresponding training cohort.

### Statistical analysis

The continuous variables were described using mean and standard deviation (SD), while the categorical variables were summarized as counts and percentages. The Mann-Whitney U-test was used to analyze the differences of numerical characteristics between patients with No.3 LNM and non-LNM. The Chi-square test and fisher’s exact test were for categorical characteristics. A two-tailed *p* < 0.05 was deemed a significant statistical difference. To evaluate inter-observer reproducibility, a second radiologist independently segmented the ROIs in 30 randomly selected cases. Intraclass correlation coefficients (ICCs) were calculated for all extracted radiomic features and model outputs. An ICC greater than 0.75 was regarded as indicative of good agreement. To assess potential multicollinearity among the four radiomic model outputs, variance inflation factors (VIFs) were calculated, with a VIF < 5 considered acceptable. All the statistical analysis was performed in R (version 3.6.0; https://www.r-project.org).

## Results

### Clinical characteristics

A total of 237, 102, and 600 patients were enrolled as the training cohort, internal validation cohort, and external validation cohort, respectively. The percentage of patients with LNM at stations No.3, 7, 8, 9 and 11 are 36.6%, 22.1%, 16.2%, 16.8% and 10.0% respectively. Detailed clinical characteristics were shown in Table 1 sorted by different sets. No significant difference in age, sex, and location of primary tumor were noted between $\:{\text{L}\text{N}\text{M}}_{\text{N}\text{o}.3}^{+}$ group and $\:{\text{L}\text{N}\text{M}}_{\text{N}\text{o}.3}^{-}$ group. (*p* > 0.05) More patients with LNM in No. 7, 8, 9 and 11 could be found in $\:{\text{L}\text{N}\text{M}}_{\text{N}\text{o}.3}^{+}$ group, suggesting a potential strong correlation between LNM in the lesser curvature and LNM in supra-pancreas. (All *p* < 0.05)

**Table 1:** Clinical characteristics of patients in the training and internal test sets

	Training set (*n* = 237)			Internal Test set (*n* = 102)		
	No.3(+)	No.3(-)	*P* value	No.3(+)	No.3(-)	*P* value
Age, $\:\stackrel{-}{x}$(SD)	55.9 (11.0)	56.4 (11.4)	0.619	56.6 (11.3)	58.6 (12.0)	0.505
Sex, n (%)			0.988			0.041
Male	56 (68.3)	106 (68.4)		34 (81.0)	42 (70.0)	
Female	26 (31.7)	49 (31.6)		8 (19.0)	18 (30.0)	
LNM in No.7, n (%)			< 0.001			< 0.001
Yes	42 (51.2)	13 (8.4)		16 (38.1)	4 (6.7)	
No	40 (48.8)	142 (91.6)		26 (61.9)	56 (93.3)	
LNM in No.8, n (%)			< 0.001			< 0.001
Yes	27 (32.9)	12 (7.7)		15 (35.7)	1 (1.7)	
No	55 (67.1)	143 (92.3)		27 (64.3)	59 (98.3)	
LNM in No.9, n (%)			< 0.001			0.002
Yes	34 (41.5)	9 (5.8)		11 (26.2)	3 (5.0)	
No	48 (58.5)	146 (94.2)		31 (73.8)	57 (95.0)	
LNM in No.11, n (%)			< 0.001			0.001
Yes	18 (22.0)	6 (3.9)		9 (21.4)	1 (1.7)	
No	64 (78.0)	149 (96.1)		33 (78.6)	59 (98.3)	

### Predictive performance of bi-regional and bi-phasic radiomic models

We first built four radiomic models (A-tumor, A-LN, V-tumor, and V-LN) to predict No.3 LNM status of patients. As shown in Additional file: Figure S1 and Figure S2, the top 20 radiomic features selected from LVQ for A-tumor, V-tumor, A-LN, and V-LN cases were listed, respectively. For bi-regional and bi-phasic radiomic models, the feature subsets consisting of the top 20 features tended to gain better performance in the training cohort (Additional file: Figure S3). Thus, we mainly built the four radiomic models based on the top 20 radiomic features, respectively, wherein extra tree, RF, LightGBM, and LRL1 were adaptively the most suitable classifiers by AutoML for the four models.

The A-tumor model yielded an AUC of 0.809 (95% confidence interval [CI], 0.718–0.901) in test cohort with a sensitivity of 61.9% at a high specificity of 86.7%. This was further verified in external test cohort by an AUC of 0.736 (95% CI, 0.691–0.781) with a sensitivity of 64.7% and a specificity of 72.3% (Table 2). The V-tumor model had an AUC of 0.817 (95% CI, 0.737–0.897), a sensitivity of 81.0%, and a specificity of 65.0% in test cohort, and achieved an AUC of 0.758 (95% CI, 0.715-0.800) with a sensitivity of 69.3% and a specificity of 64.9% in external test cohort. As to the A-LN model and V-LN model, both showed performance degradation compared to tumor-based models, but still had AUCs over about 0.700 in test cohorts. Of note, the repeated random subsampling validation results on Nanfang Hospital dataset were summarized in Additional file: Table S1 and Figure S4, indicating the stability of our methods. In addition, inter-observer reproducibility analysis showed that 83.8% of the radiomic features used in model construction had ICCs greater than 0.75, indicating good feature-level consistency. The predicted outputs of all four radiomic models also demonstrated high robustness, with ICCs all exceeding 0.80. These results confirm the overall reliability and reproducibility of our segmentation process and modeling pipeline.

**Table 2:** Model performance in predicting no.3 lymph node metastasis status

Models	Cohorts	AUC (95% CI)	SEN (%)	SPE (%)	ACC (%)
A-tumor model	Training	0.774 (0.714–0.833)	59.8	79.4	72.6
	Test	0.809 (0.718–0.901)	61.9	**86.7**	76.5
	External test	0.736 (0.691–0.781)	64.7	72.3	70.3
V-tumor model	Training	0.826 (0.773–0.879)	**82.9**	66.5	72.2
	Test	0.817 (0.737–0.897)	81.0	65.0	71.6
	External test	0.758 (0.715-0.800)	**69.3**	64.9	66.0
A-LN model	Training	0.794 (0.735–0.854)	67.1	**81.9**	76.8
	Test	0.715 (0.614–0.817)	61.9	65.0	63.7
	External test	0.699 (0.649–0.749)	45.8	**83.9**	74.2
V-LN model	Training	0.742 (0.674–0.809)	62.2	77.4	72.2
	Test	0.697 (0.593–0.802)	57.1	75.0	67.6
	External test	0.653 (0.604–0.703)	56.2	67.8	64.8
Hybrid model	Training	**0.900 (0.860–0.939)**	78.0	81.3	**80.2**
	Test	**0.847 (0.770–0.924)**	**83.3**	76.7	**79.4**
	External test	**0.833 (0.800-0.867)**	63.4	81.2	**76.7**

NOTE. Bold values represent the best performance. AUC, area under the curve; CI, confidence interval; SEN, sensitivity; SPE, specificity; ACC, accuracy

### Performance evaluation of hybrid model in predicting metastasis status of lesser curvature LN station (No.3)

**Fig. 2:**
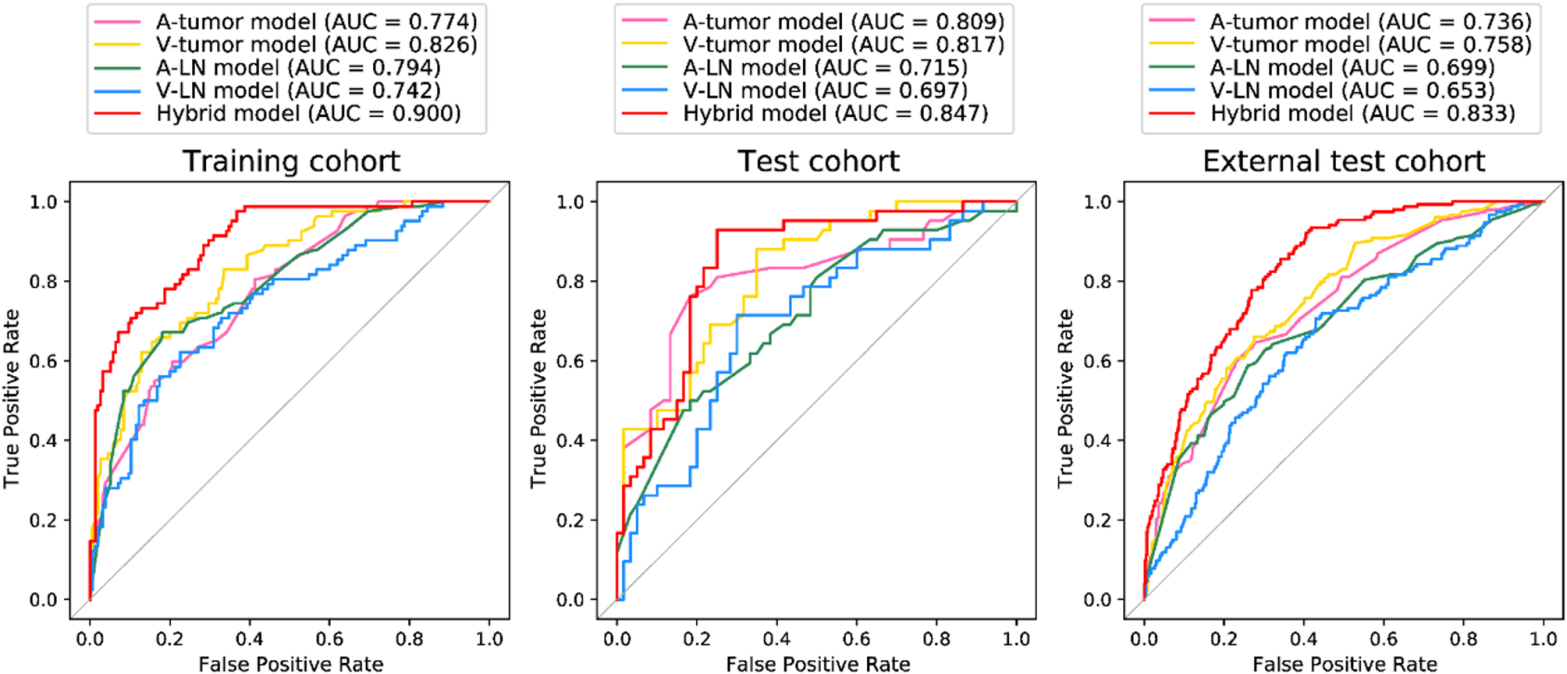
Receiver operating characteristic curves for radiomic models in predicting No.3 lymph node metastasis. AUC, area under the curve

VIF analysis showed low multicollinearity among the four radiomic model outputs (A-tumor: 3.25, V-tumor: 3.45, A-LN: 1.29, V-LN: 1.11), supporting their joint use in a hybrid model. We integrated the predictions of four bi-regional and bi-phasic radiomic models to build a hybrid model (Additional file: Table S2), which yielded substantially higher performance with AUCs of 0.847 (95% CI, 0.770–0.924) and 0.833 (95% CI, 0.800-0.867) in the two test cohorts (Fig. 2). In addition, we conducted repeated random subsampling validation. Additional file: Figure S5 shows the ROC curves for the hybrid model in five-time repeated random subsampling validation in training and test cohort, suggesting the robustness of our as-developed model. Notably, the hybrid model could generate fewer false negative cases and scored the highest in AUC and accuracy, which could mitigate the probable severe consequences brought by misdiagnosis.

**Fig. 3:**
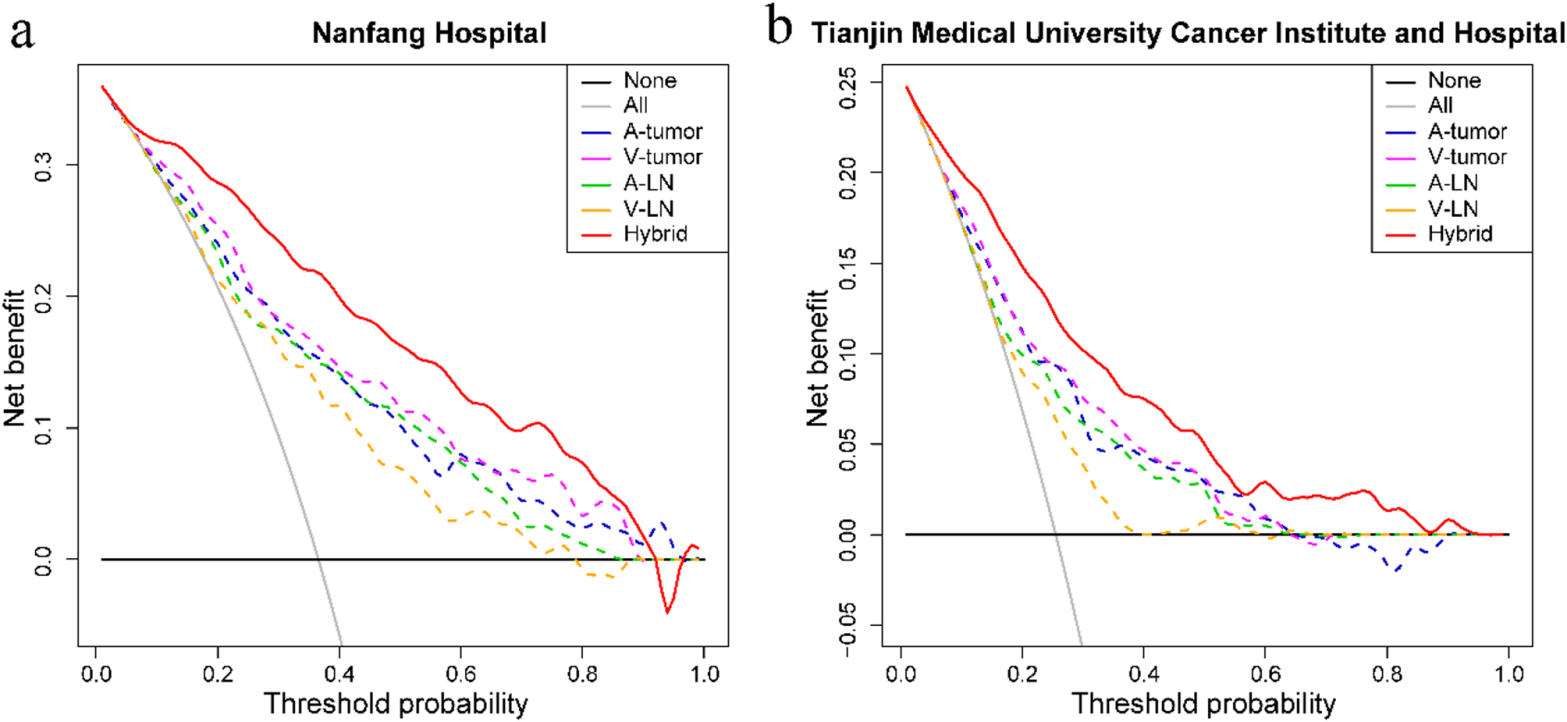
Decision curve analysis for hybrid model as well as A-tumor, V-tumor, A-LN, and V-LN models on datasets from the two hospitals

The decision curves illustrated that using the hybrid model to predict No.3 LNM status could provide incremental clinical benefit from the threshold probability of 0 to 90%, compared with the intervention-for-all and intervention-for-none schemes as well as the four bi-regional and bi-phasic radiomic models (Fig. 3). The hybrid model showed a dominant role in No.3 LNM status prediction.

### The efficacy of hybrid model in predicting metastasis status of Supra-pancreatic LN stations (No. 7, 8, 9, 11)

In general, gastric cancer patients with No.3 LNM are more likely to have LNM in No.7, 8, 9, and 11, which corresponds to our clinical data (Table 1). Besides, the LN-ROI included the supra-pancreatic area (No.7, 8, 9, and 11) but less subhepatic area (No. 8). Thus, to verify that the model demonstrates good generalization performance, we further explored whether the hybrid model for No.3 LNM prediction could remain effective on predicting metastasis status of supra-pancreatic LN stations (No.7, 8, 9, and 11). As shown in Table 3, the potential generalization ability of the hybrid model to predict LNM in other LN stations can be proved by these mildly high AUCs with a range from 0.678 to 0.761 in two test cohorts. In addition, the hybrid model showed a slightly worse performance in the prediction of LNM in No.8 (nodes near proper hepatic artery) with AUCs of 0.683 (95%CI, 0.554–0.812) and 0.678 (95%CI, 0.615–0.741) respectively in two test cohorts, compared to No.7 (Left Gastric nodes), No.9 (Celiac nodes) and No.11 (Nodes near spleen artery).

**Table 3:** The efficacy of hybrid model predictions in analyzing metastasis status of supra-pancreatic LN stations (No.7, 8, 9, 11)

Stations	Cohorts	No. of patients	AUC (95% CI)	SEN (%)	SPE (%)	ACC (%)
No.7	Training	237/237, 55/237	0.800 (0.740–0.861)	78.2	72.5	73.8
	Test	102/102, 20/102	0.757 (0.660–0.855)	90.0	62.2	67.6
	External test	535/600, 91/535	0.683 (0.622–0.745)	49.5	73.9	69.7
No.8	Training	237/237, 39/237	0.770 (0.697–0.844)	74.4	72.7	73.0
	Test	102/102, 16/102	0.683 (0.554–0.812)	56.3	62.8	61.8
	External test	540/600, 90/540	0.678 (0.615–0.741)	48.9	79.3	74.3
No.9	Training	237/237, 43/237	0.751 (0.670–0.832)	72.1	76.3	75.5
	Test	102/102, 14/102	0.761 (0.654–0.867)	64.3	68.2	67.6
	External test	541/600, 83/541	0.680 (0.617–0.743)	43.4	82.5	76.5
No.11	Training	237/237, 24/237	0.695 (0.581–0.809)	79.2	56.3	58.6
	Test	102/102, 10/102	0.758 (0.639–0.876)	90.0	51.1	54.9
	External test	416/600, 55/416	0.687 (0.618–0.756)	69.1	61.5	62.5

NOTE. The third column shows No. of patients with LNM status labels/No. of all the patients, No. of patients with LNM/No. of patients with LNM status labels. AUC, area under the curve; CI, confidence interval; SEN, sensitivity; SPE, specificity; ACC, accuracy

## Discussion

In this work, we analyzed features related to LNM in both the primary lesion and the lesser curvature region across different phases, and built a new bi-regional and bi-phase radiomic model to define LNM in lesser curvature LN stations. In addition to its clinical relevance, the lesser curvature and the supra-pancreatic area were selected as ROIs due to their relative stability across different patients’ CT imaging, attributed to the tensile strength of the hepatoduodenal ligament and the hepatogastric ligament [8]. And the five-time repeated random subsampling validation for our hybrid model demonstrated high predictive capability and reproducibility for LNM in the lesser curvature and showed potential for extending the prediction to LNM in the supra-pancreatic region.

Several studies have focused on the prediction of LNM in GC. However, these previous studies delineated primary tumor as ROIs, emphasizing the effect of tumor cells while ignoring the lymphatic tissue itself. In our earlier work, we developed a deep learning radiomic nomogram (DLRN) based on tumor region segmentation to discriminate the number of metastatic LNs in locally advanced gastric cancer, achieving AUC values between 0.797 and 0.821 [30]. But predicting the total number of metastatic LNs highly relies on the examination of dissected LNs and fails to capture the anatomic features of LNM. To address this limitation, *Jin et al.*. developed a deep learning system specifically designed to predict LNM in individual LN stations, obtaining AUC values ranging from 0.856 to 0.893 [31]. And another study that focused on the prediction of LNM in the lesser curvature reported AUC values from 0.705 to 0.823 [32]. Compared to these previous works that performed on primary 2D tumor region segmentation using single-phase CT images [30, 33, 34], our model was performed on four 3D-ROIs derived from bi-phase and bi-region CT images and showed AUC values of 0.833–0.847. Our hybrid model achieved higher AUC values for the prediction of LNM in the lesser curvature, indicating superior diagnostic performance for LNM in individual LN stations. Consequently, we established a hybrid model with robust predictive power based on bi-phasic and bi-regional 3D ROIs.

Moreover, the predictive value of our model did not rely on clinical characteristics, such as the location of primary tumor and TNM stage, suggesting that the image features we captured and used in the hybrid model might be associated with specific biological behaviors of the tumor. In our hybrid model, the highest coefficient was found in A-LN model, followed by the V-tumor model. This finding implies that LNM is associated with both primary lesion and the mesogastric LN-containing adipose tissue isolated by the fascia. This corresponds to the “seed and soil” theory, which proposes that the interaction between primary tumor cells (the ‘seed’) and specific organ microenvironments (the ‘soil’) is critical for the process of metastasis [35]. As is well known, the arterial phase is more sensitive for identifying lesions with rich blood supply, while detection for lesions with less blood supply is much easier in the venous phase. Given that GC mainly involves vascular occlusion and can be easily distinguished from normal tissue in the venous phase, it might explain why the venous phase of primary tumor achieved a better predictive performance. Nevertheless, the arterial phase is more sensitive for LN region, indicating tumor cells (the ‘seed’) would be easier to move to the region (the ‘soil’) with rich blood supply.

For detailed image features, the gray level run length matrix (GLRLM) and gray level size zone matrix (GLSZM) in tumor region appears to be pivotal in LNM. It suggests that some strips of tumor, capillaries or fiber structure, play an important role in LNM. But it does not imply that the surrounding microenvironment is without influence. The features of the tissues included in LN region of our model reflect on the gray level intensity, and the fineness and the coarseness of texture, indicating that tumor cells tend to metastasize to the areas that are less uniform and highly heterogenous. This finding was similar to what we observed in occult peritoneal metastasis [22]. 

The direct delineation of lymph nodes in CT imaging was technically constrained by their limited visibility. To address this, we adopted an alternative approach by delineating the entire lesser curvature region, thereby capturing radiomic features of tumor-surrounding tissues that may reflect potential LNM. Specifically, the inherent resolution limitations of CT imaging in visualizing small peritumoral vessels and tissues necessitated reliance on discernible anatomical landmarks (e.g., stomach, liver, pancreas, celiac trunk, splenic artery, and left gastric artery) for defining the LN ROI. It is inevitable to include supra-pancreatic lymph node stations (No.7, No.8, No.9, No.11) in addition to the lesser curvature station (No.3). However, the clear visualization of the liver and proper hepatic artery on CT led to partial exclusion of station No. 8, which may account for its reduced predictive performance in our model. Generalization of this model to the supra-pancreatic area demonstrated its potential clinical utility, albeit with station-specific variations. These findings suggest that region-based radiomic analysis, despite its inability to directly localize individual LNs, may serve as a surrogate for identifying high-risk metastatic patterns.

There’re some limitations in our study. First, we did not incorporate patients’ clinical characteristics and pathological information into our model, which hinders correlating radiomic features with exact tumor biological behaviors and deciphering more detailed insights. Besides, as a retrospective study, selection bias remains hard to remove. The absence of external validation in Western populations limits the generalizability of our findings. Importantly, while neoadjuvant therapy is known to induce profound morphological alterations, the exclusion of patients receiving such treatment restricts our model’s applicability to larger cohorts. In addition, our present study just used handcraft features although the robustness of our pipeline was supported by high ICC across segmentation, and future research should explore automated segmentation techniques to enhance this model.

In conclusion, LNM in GC impacts the selection of perioperative treatment strategies. To address this issue, we developed a bi-phasic and bi-regional radiomic model based on the primary lesion and surrounding LN-containing tissue to define LNM to the lesser curvature and supra-pancreatic LN stations. This model showed great predictive capability, reproducibility, and generalizability for LNM in the lesser curvature, and has the potential to serve as a valuable tool for individualized treatment of GC.

## Electronic supplementary material

Below is the link to the electronic supplementary material.

Supplementary Material 1

## Data Availability

No datasets were generated or analysed during the current study.
