# Supplementary material for: Bi-regional and bi-phasic automated machine learning radiomics for defining metastasis to lesser curvature lymph node stations in gastric cancer
Source: Cancer Imaging. 2025 Jun 8;25:71. doi: 10.1186/s40644-025-00891-z (PMC12147310; doi:10.1186/s40644-025-00891-z)

**Supplementary Materials**

**Supplementary Tables**

**Table S1.** Average model performance of five-time repeated random subsampling validation.

**Table S2.** The calculation formula for hybrid model.

**Supplementary Figures**

**Figure S1.** The top 20 radiomic features selected from LVQ for A-tumor and V-tumor cases based on the training cohort. LVQ, learning vector quantization; LNM, lymph node metastasis.

**Figure S2.** The top 20 radiomic features selected from LVQ for A-LN and V-LN cases based on the training cohort. LVQ, learning vector quantization; LNM, lymph node metastasis.

**Figure S3.** Performance of models by fitting the top 5, 10, 15, and 20 radiomic features from LVQ for A-tumor, V-tumor, A-LN, and V-LN cases. The training and test cohorts were generated from repeated random subsampling validation based on the Nanfang Hospital dataset.

**Figure S4.** ROC curves for A-tumor, V-tumor, A-LN, and V-LN models in five-time repeated random subsampling validation. The red line highlighted the mean ROC curves, and the gray regions represented shading marks proportional to standard deviations. ROC, receiver operating characteristic; AUC, area under the curve.

**Figure S5.** ROC curves for hybrid model in five-time repeated random subsampling validation. The red line highlighted the mean ROC curves, and the gray regions represented shading marks proportional to standard deviations. ROC, receiver operating characteristic; AUC, area under the curve.

**Table S1.** Average model performance of five-time repeated random subsampling validation.

| **Cohorts /**  **Evaluation metrics** | | **A-tumor model** | **V-tumor model** | **A-LN model** | **V-LN model** | **Hybrid model** |
| --- | --- | --- | --- | --- | --- | --- |
| Training cohort | AUC | 0.797±0.014 | 0.817±0.017 | 0.806±0.009 | 0.772±0.023 | **0.907±0.014** |
|  | Accuracy | 0.747±0.022 | 0.740±0.011 | 0.759±0.019 | 0.729±0.018 | **0.834±0.022** |
|  | Sensitivity | 0.671±0.037 | 0.744±0.058 | 0.725±0.039 | 0.699±0.050 | **0.817±0.031** |
|  | Specificity | 0.788±0.030 | 0.739±0.047 | 0.778±0.049 | 0.743±0.035 | **0.843±0.020** |
| Test cohort | AUC | 0.791±0.015 | 0.784±0.021 | 0.725±0.013 | 0.702±0.006 | **0.832±0.011** |
|  | Accuracy | 0.716±0.035 | 0.702±0.017 | 0.672±0.035 | 0.653±0.036 | **0.751±0.027** |
|  | Sensitivity | 0.661±0.106 | **0.728±0.061** | 0.677±0.054 | 0.633±0.077 | 0.725±0.057 |
|  | Specificity | 0.764±0.078 | 0.690±0.039 | 0.665±0.065 | 0.675±0.102 | **0.765±0.023** |

NOTE. The model performances were presented as average ± standard deviation. AUC, area under the curve.

**Table S2.** The calculation formula for hybrid model.

| **Index** | **Coefficients** | ***P* values** |
| --- | --- | --- |
| A-tumor model prediction | 3.0519 | 0.0976 |
| V-tumor model prediction | 5.7315 | 0.0001* |
| A-LN model prediction | 6.3083 | < 0.0001* |
| V-LN model prediction | 3.2366 | 0.0047* |
| Intercept | -7.3770 | < 0.0001* |

**Figure S1.** The top 20 radiomic features selected from LVQ for A-tumor and V-tumor cases based on the training cohort. LVQ, learning vector quantization; LNM, lymph node metastasis.

**
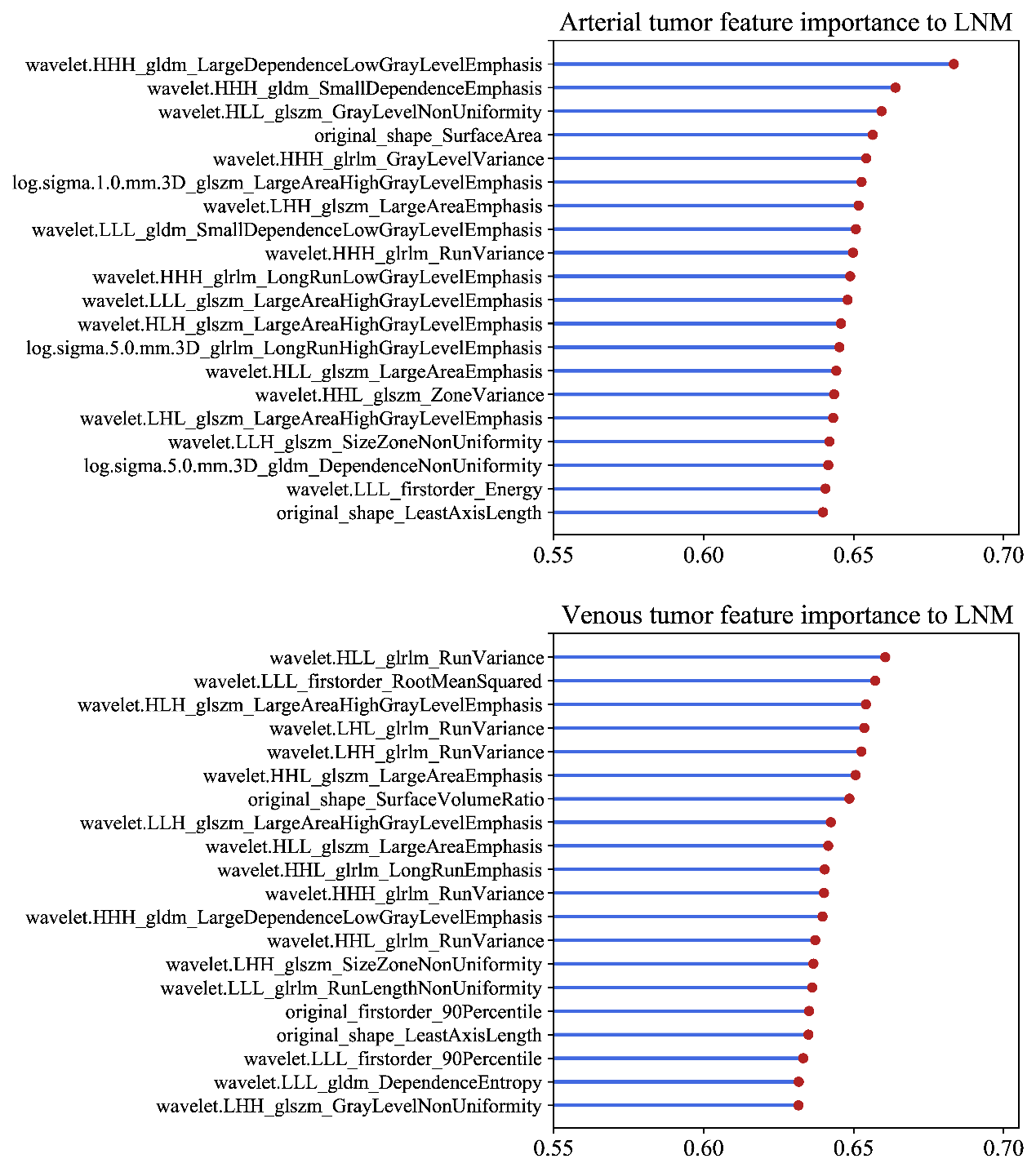
**

**Figure S2.** The top 20 radiomic features selected from LVQ for A-LN and V-LN cases based on the training cohort. LVQ, learning vector quantization; LNM, lymph node metastasis.

**
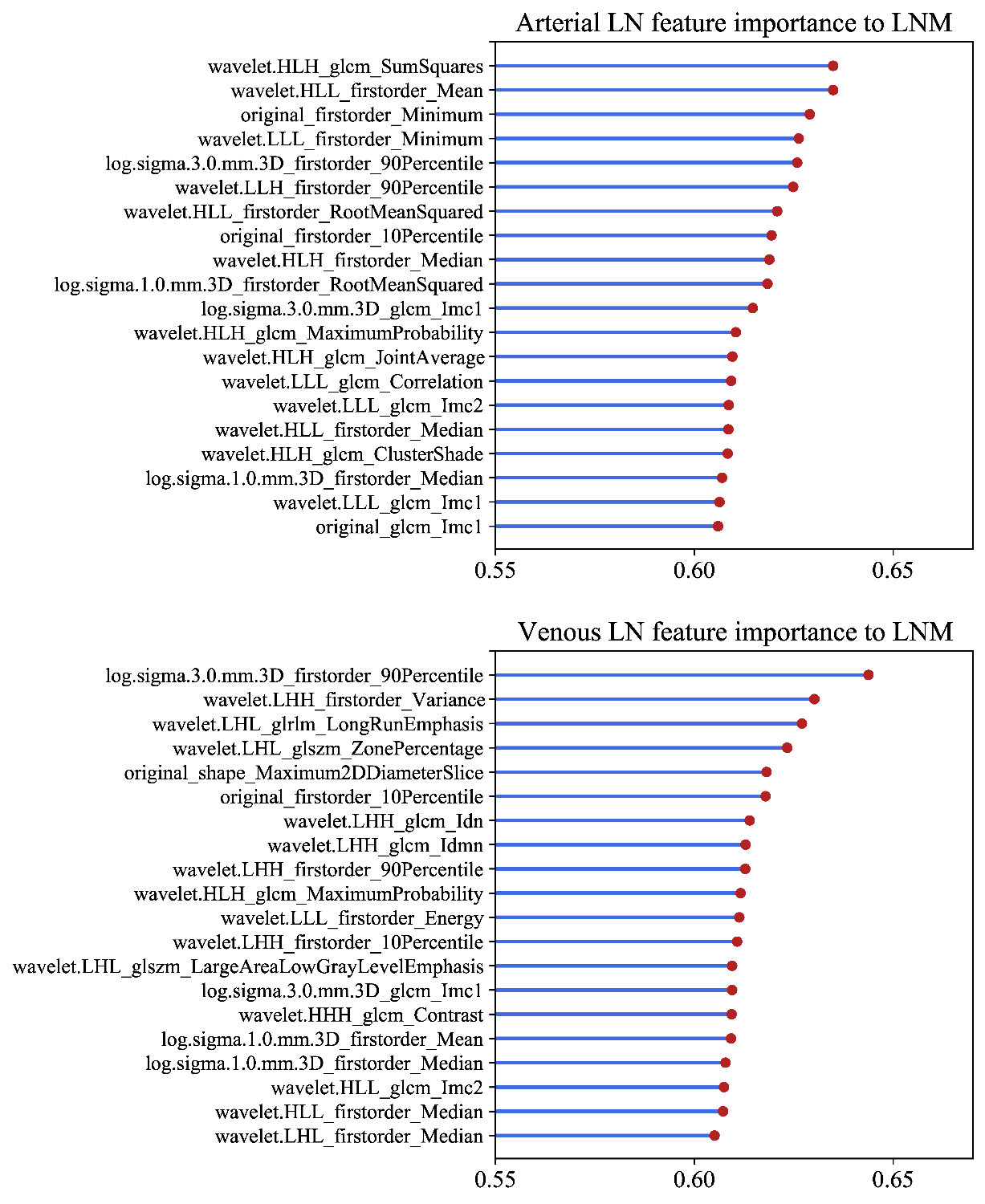
**

**Figure S3.** Performance of models by fitting the top 5, 10, 15, and 20 radiomic features from LVQ for A-tumor, V-tumor, A-LN, and V-LN cases. The training and test cohorts were generated from repeated random subsampling validation based on the Nanfang Hospital dataset. *represents the highest training AUC. LVQ, learning vector quantization; LN, lymph node; AUC, area under the curve.

**
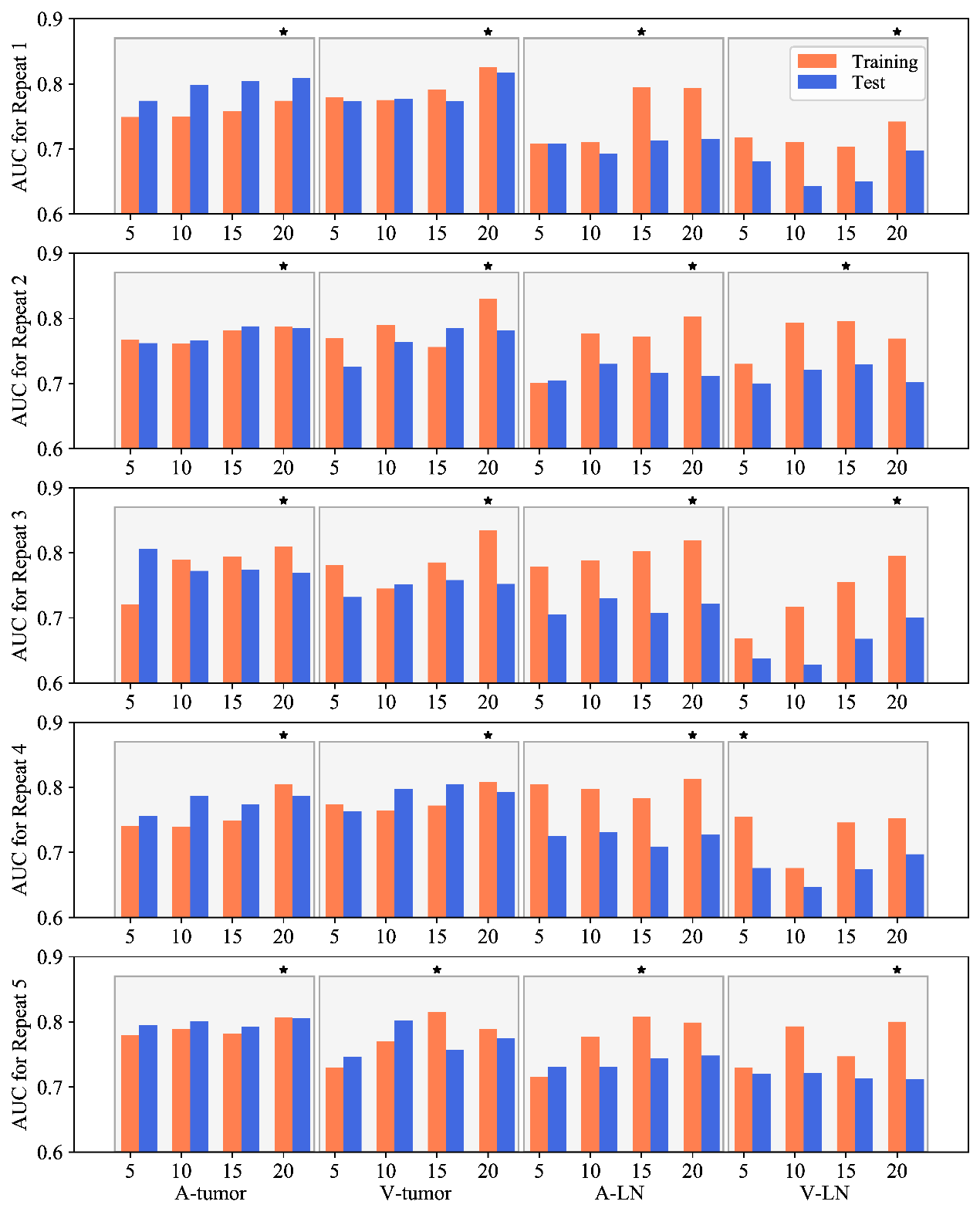
**

**Figure S4.** ROC curves for A-tumor, V-tumor, A-LN, and V-LN models in five-time repeated random subsampling validation. The red line highlighted the mean ROC curves, and the gray regions represented shading marks proportional to standard deviations. ROC, receiver operating characteristic; AUC, area under the curve.


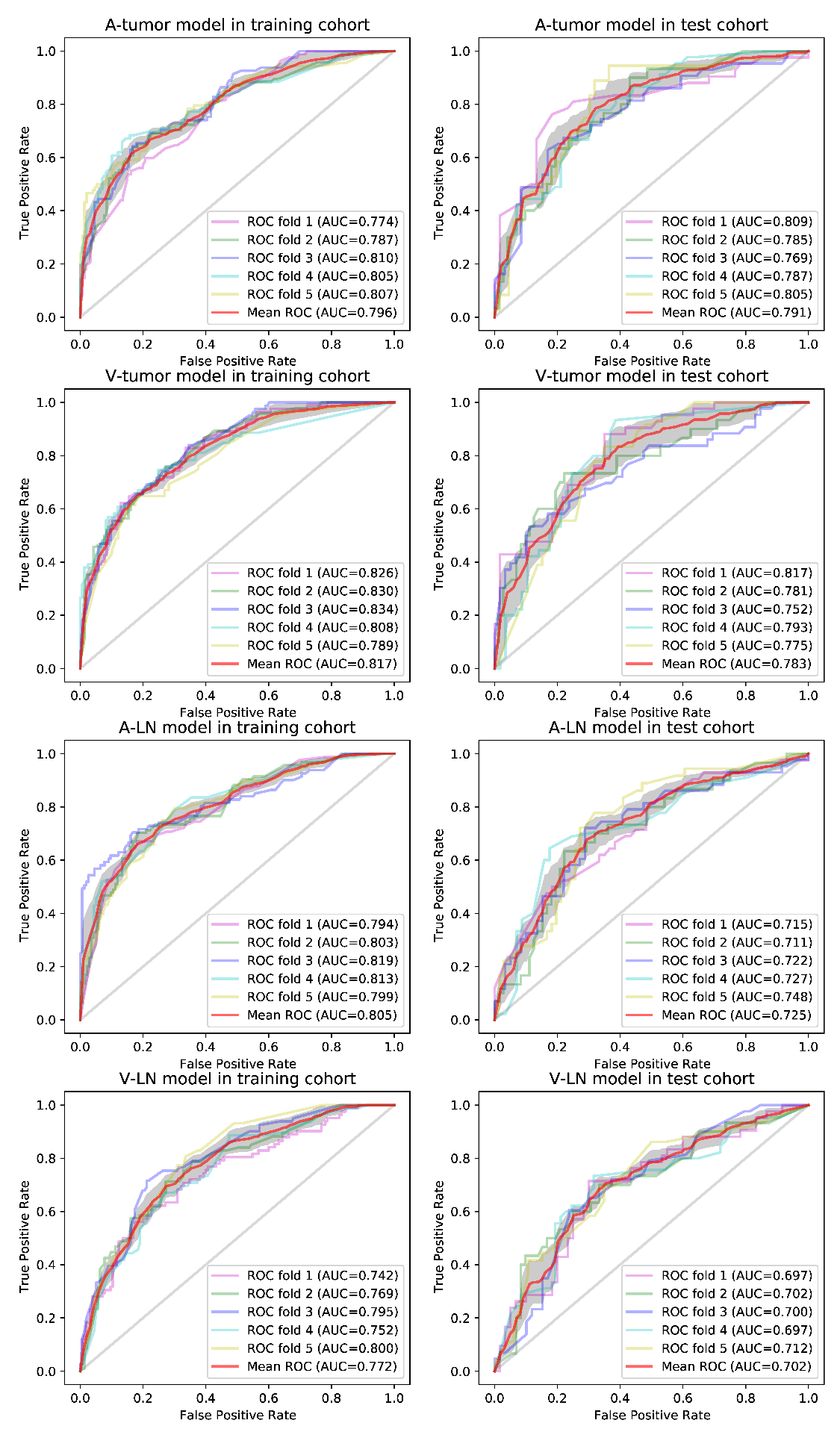


**Figure S5.** ROC curves for hybrid model in five-time repeated random subsampling validation. The red line highlighted the mean ROC curves, and the gray regions represented shading marks proportional to standard deviations. ROC, receiver operating characteristic; AUC, area under the curve.


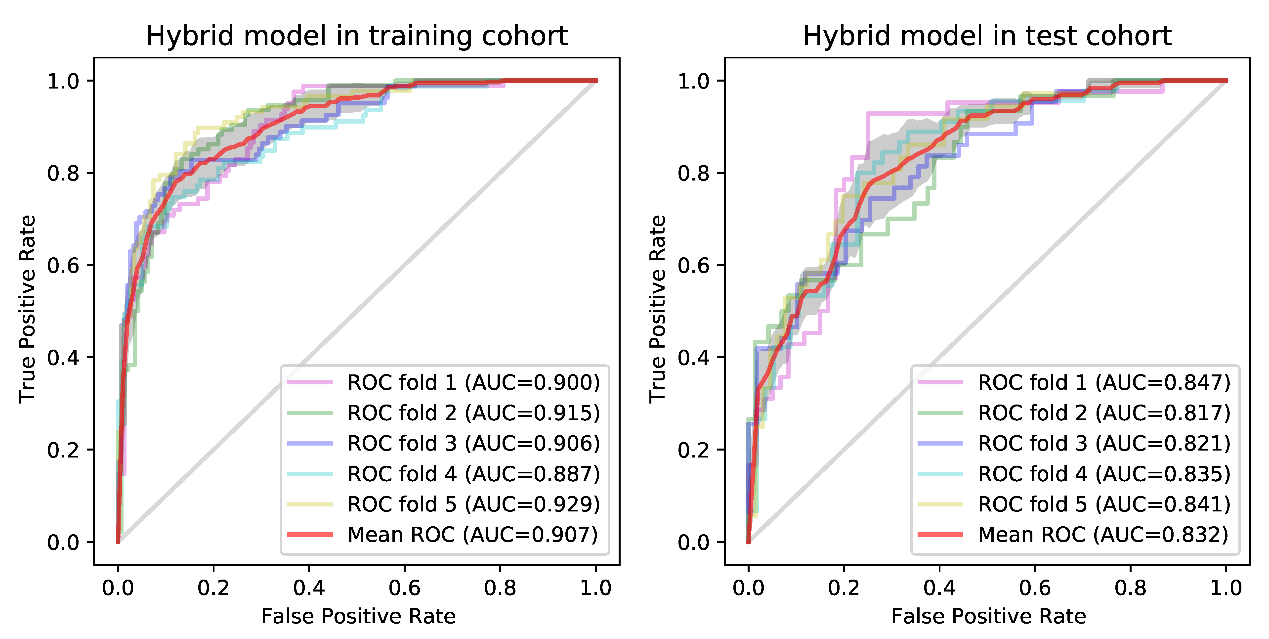

Supplement: Supplementary file 1 — Supplementary Material 1 [file 40644_2025_891_MOESM1_ESM.docx]
